# Tumor Regression Following Neoadjuvant Therapy as a Determinant of Surgical Resectability in Stage IIIA Non-small Cell Lung Cancer: A Comparative Clinical Study

**DOI:** 10.7759/cureus.107352

**Published:** 2026-04-19

**Authors:** Safet Musanovic, Ilijaz Pilav, Alen Pilav, Orhan Custovic, Zlatan Zvizdic

**Affiliations:** 1 Thoracic Surgery, Klinički Centar Univerziteta u Sarajevu, Sarajevo, BIH; 2 Pediatric Surgery, Klinički Centar Univerziteta u Sarajevu, Sarajevo, BIH

**Keywords:** neoadjuvant therapy, stage iiia nsclc, surgical outcomes, tnm downstaging, tumor regression grade

## Abstract

Background

This study explored whether tumor regression following neoadjuvant therapy can be used as a reliable indicator of surgical operability in patients with stage IIIA non-small cell lung cancer (NSCLC).

Methods

A retrospective cohort analysis was performed, including patients with stage IIIA NSCLC treated at a tertiary thoracic surgery center. Patients were categorized according to treatment approach: induction therapy followed by surgery or primary surgical management. Treatment response was assessed using imaging findings, pathological staging changes, residual tumor burden, and lymph node status. Surgical feasibility and perioperative outcomes were evaluated. Statistical significance was defined at p<0.05.

Results

Patients receiving induction therapy demonstrated greater tumor reduction, higher rates of mediastinal nodal regression, and more frequent complete pathological response. Complete (R0) resection was achieved more often in this group. Tumor regression and nodal response were identified as independent predictors of surgical feasibility. Postoperative complication rates and mortality did not differ significantly between groups.

Conclusion

Tumor response after neoadjuvant therapy is closely associated with surgical operability in stage IIIA NSCLC. Response-based selection may improve resectability without increasing perioperative risk.

## Introduction

Non-small cell lung cancer (NSCLC) remains one of the leading causes of cancer-related mortality worldwide. A considerable proportion of patients present with locally advanced disease, most commonly stage IIIA, which is characterized by variability in tumor size, lymph node involvement, and biological behavior [1,2].

The management of stage IIIA NSCLC requires a multimodal approach integrating systemic therapy, radiotherapy, and surgical intervention. The selection of appropriate treatment strategies depends on disease extent and patient-related factors, making therapeutic decision-making complex [3,4].

Neoadjuvant therapy has increasingly been incorporated into treatment protocols to reduce tumor burden and improve the likelihood of complete surgical resection. In selected cases, induction therapy may convert initially borderline or unresectable tumors into operable disease. However, the benefit of surgery is strongly influenced by the degree of response to treatment [5-7].

Various parameters, including radiological response, pathological downstaging, and lymph node status, have been investigated as indicators of treatment efficacy. Despite this, clear criteria for determining operability following neoadjuvant therapy remain insufficiently defined [8,9].

The aim of this study was to evaluate whether tumor regression after induction therapy can be used as a clinically relevant criterion for assessing operability and guiding treatment decisions in patients with stage IIIA NSCLC.

## Materials and methods

This was retrospective study conducted at Klinički Centar Univerziteta u Sarajevu (Clinical Center University of Sarajevo), Sarajevo, Bosnia and Herzegovina, between January 2012 and December 2023. The study was approved by the Ethics Committee of the Clinical Center University of Sarajevo (approval number: 51-45-1-46289/23). Written informed consent was obtained from all patients included in the study.

Study participants

Patients with histologically confirmed NSCLC, clinical stage IIIA disease, surgical treatment with curative intent, and complete clinical, radiological, operative, and pathological data, were included in the study. Exclusion criteria were: small cell lung carcinoma, stage IIIB or IV disease, previous lung cancer treatment, incomplete medical records, and palliative surgical procedures.

All patients were reviewed by a multidisciplinary tumor board, and staging was performed according to the eighth edition of the TNM classification [1].

Treatment groups

Patients were divided into two groups based on treatment strategy. The first group received neoadjuvant therapy followed by surgical resection (Study group), while the second group underwent primary surgery without prior systemic treatment (Control group). Treatment decisions were based on tumor characteristics, nodal involvement, functional status, and multidisciplinary consensus.

Preoperative evaluation

All patients underwent comprehensive preoperative assessment, including clinical evaluation, pulmonary function testing, contrast-enhanced computed tomography of the thorax and upper abdomen, and brain imaging. Functional imaging with PET/CT was performed when available. Mediastinal staging was carried out using endobronchial ultrasound-transbronchial needle aspiration (EBUS-TBNA), mediastinoscopy, or both when indicated.

Neoadjuvant therapy

Induction therapy consisted of platinum-based combination chemotherapy, with or without radiotherapy, according to institutional protocols. Radiotherapy was delivered using modern conformal techniques. Response to treatment, including reassessment of mediastinal lymph nodes, was primarily evaluated using cross-sectional imaging (contrast-enhanced CT and/or PET-CT, when available). In cases where imaging findings were inconclusive or where residual nodal disease required further clarification, invasive mediastinal restaging was performed using EBUS-TBNA and/or mediastinoscopy. Surgery was scheduled after an appropriate interval (four to six weeks) following completion of neoadjuvant therapy.

Surgical procedure

All patients underwent anatomical lung resection, including lobectomy, bilobectomy, or pneumonectomy, depending on tumor extent. Systematic mediastinal lymph node dissection was performed in all cases.

Complete (R0) resection was defined as the absence of microscopic residual tumor at the resection margins, confirmed by standard histopathological examination of surgical specimens, including bronchial, vascular, and parenchymal margins, as well as systematic evaluation of resected lymph nodes. In cases of suspected tumor proximity to resection margins, additional histopathological assessment was performed. Deaths occurring within the first 30 postoperative days, as well as those occurring prior to discharge from the initial hospital admission, were classified as operative mortality.

Pathological evaluation

Resected specimens were analyzed by experienced pathologists. Tumor regression was evaluated based on residual viable tumor, pathological downstaging, and lymph node status (ypN). Major pathological response was defined as ≤10% viable tumor cells, while a complete response indicated no residual viable tumor.

Outcome measures

The primary outcome was the rate of complete surgical resection (R0). Secondary outcomes included type of resection, postoperative complications, perioperative mortality, pathological response, and nodal downstaging.

Nodal downstaging was defined as a change in mediastinal lymph node status from clinical stage cN2 to ypN0-ypN1 following neoadjuvant therapy. Initial staging was performed using a combination of imaging and invasive modalities (CT, PET/CT when available, EBUS-TBNA, and/or mediastinoscopy), in accordance with current guidelines.

After neoadjuvant therapy, restaging was performed using CT and/or PET/CT, with selective use of invasive methods when clinically indicated. Final nodal status was determined based on histopathological examination of resected lymph nodes following systematic mediastinal lymphadenectomy.

Statistical analysis

Statistical analyses were performed using appropriate parametric and non-parametric tests. The Chi-square test or Fisher’s exact test, as appropriate, was used for categorical variables. Continuous variables were analyzed using the Student’s t-test for normally distributed data or the Mann-Whitney U test for non-parametric distributions. Continuous variables are presented as mean ± standard deviation (SD) or median (interquartile range (IQR)), depending on data distribution, while categorical variables are presented as frequencies and percentages.

Multivariable logistic regression analysis was used to identify independent predictors of surgical operability. The final model included two variables: tumor regression and nodal downstaging. The events-per-variable (EPV) ratio was adequate for stable model estimation, thereby minimizing the risk of overfitting. A p-value <0.05 was considered statistically significant. All analyses were performed using IBM SPSS Statistics for Windows, Version 26.0 (IBM Corp., Armonk, New York, United States).

## Results

A total of 80 patients were included and equally distributed between the two treatment groups. Baseline characteristics were comparable, with no significant differences observed. (Table 1).

**Table 1:** Baseline characteristics of patients

Variable	Neoadjuvant group (n=40)	Control group (n=40)
Mean age (years)	62	63
Male sex, n (%)	30 (75%)	28 (70%)
Female sex, n (%)	10 (25%)	12 (30%)

Tumor response parameters, including partial response, stable disease, progressive disease, major pathological response, and complete pathological response, were assessed exclusively in the neoadjuvant group, as these outcomes were not applicable to the control group. Nodal downstaging (cN2 to ypN0-N1) was evaluated in both groups and demonstrated a significantly higher rate in the neoadjuvant group compared to the control group. (Table 2).

**Table 2:** Treatment response and nodal downstaging in the neoadjuvant and control groups

Outcome	Neoadjuvant group, n (%)	Control group, n (%)	p-value (Fisher's test)
Partial response	26 (65%)	-	-
Stable disease	10 (25%)	-	-
Progressive disease	4 (10%)	-	-
Major pathological response	14 (35%)	-	-
Complete pathological response	6 (15%)	-	-
Nodal downstaging (N2 → N0–1)	22 (55%)	4 (10%)	<0.001

Complete (R0) resection was achieved more often in patients treated with induction therapy. The extent of surgical procedures was similar between groups.

Postoperative complications and perioperative mortality rates were comparable, indicating that neoadjuvant therapy did not increase surgical risk. Thirty-day postoperative mortality was 2.5% (one patient) in the neoadjuvant group and 5% (two patients) in the control group, without a significant difference (p=0.55). (Table 3.)

**Table 3:** Surgical outcome

Outcome	Neoadjuvant group, n (%)	Control group, n (%)	p-value
R0 resection	36 (90%)	30 (75%)	0.04
Postoperative complications	11 (27.5%)	12 (30%)	0.79
30-day mortality	1 (2.5%)	2 (5%)	0.55

Surgical procedures and completeness of resection

Anatomical pulmonary resection was achieved in all included patients. Within the neoadjuvant cohort, lobectomy represented the most frequently performed procedure (28 patients, 70%), followed by pneumonectomy in seven patients (17.5%) and bilobectomy in five cases (12.5%). In contrast, the control group showed a slightly different distribution, with lobectomy performed in 25 patients (62.5%), pneumonectomy in nine patients (22.5%), and bilobectomy in six patients (15%), without a statistically significant difference between groups (p=0.62).

**Figure 1:**
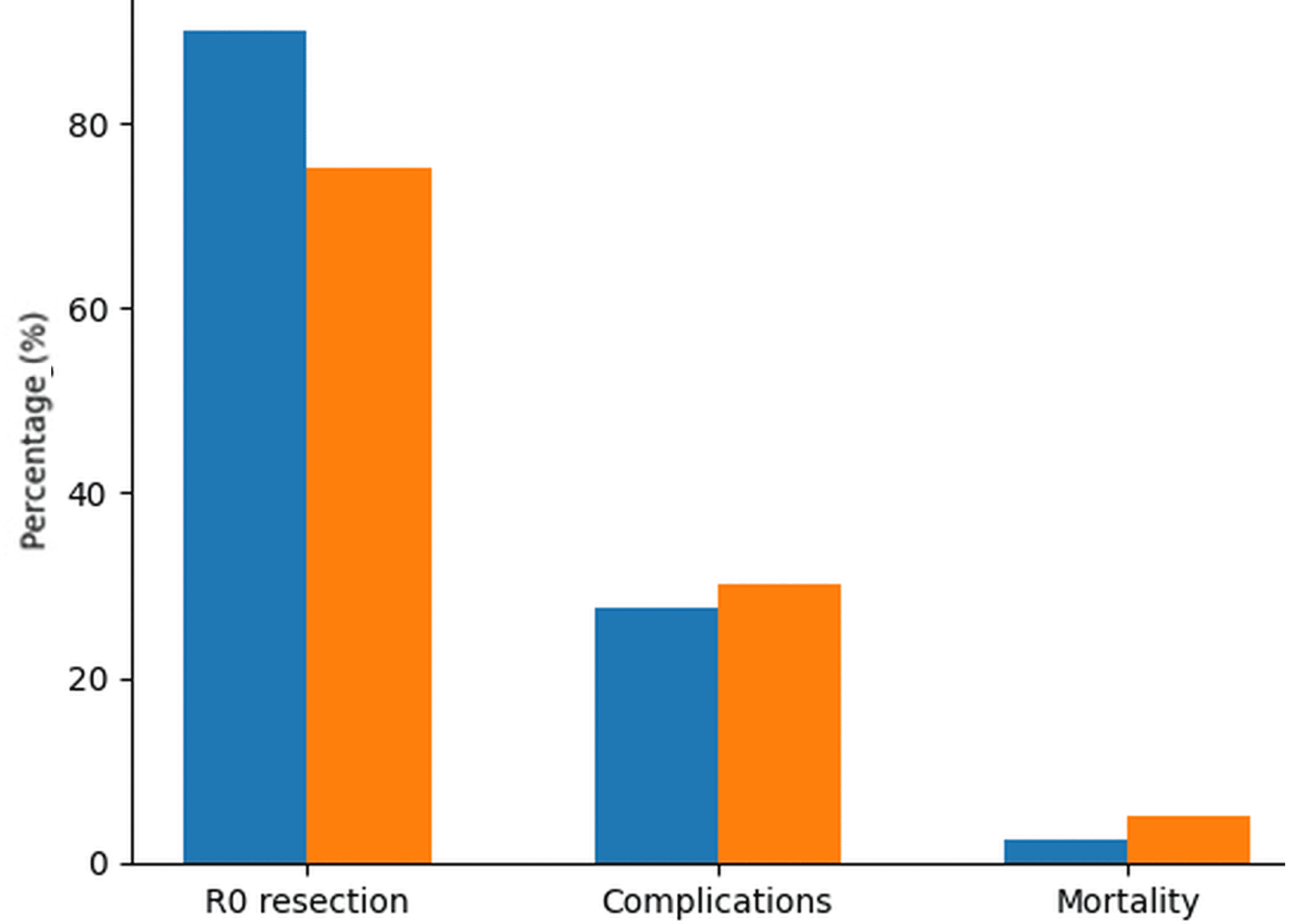
Comparison of surgical cutcomes Bar graph comparing key surgical endpoints in patients with stage IIIA NSCLC. The neoadjuvant group (n=40) showed a higher rate of R0 resection than the control group (n=40), indicating improved tumor resectability after induction therapy. Postoperative complication rates were comparable between groups. Mortality remained low in both cohorts. These findings suggest that neoadjuvant treatment increases the likelihood of complete resection without additional perioperative risk. NSCLC: non-small cell lung cancer

A complete resection with negative margins (R0) was more frequently accomplished in patients who underwent induction therapy, being achieved in 36 cases (90%), compared to 30 patients (75%) in the control group. This difference reached statistical significance, indicating improved surgical operability following neoadjuvant treatment (p=0.04) (Figure 1).

Nodal downstaging and residual tumor burden

**Figure 2:**
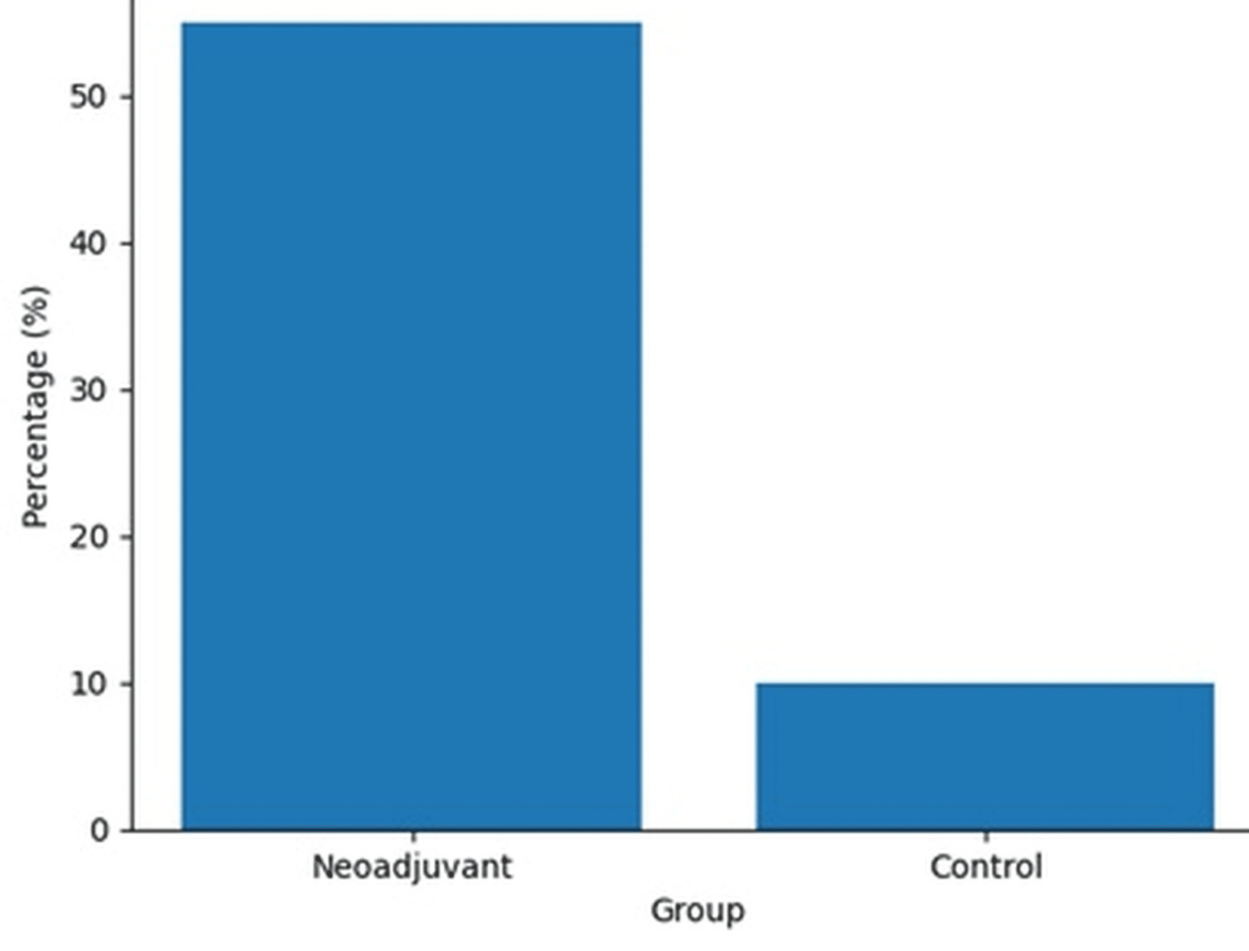
Mediastinal nodal downstaging (N2-N0-1) A significantly greater proportion of patients in the neoadjuvant group achieved mediastinal nodal downstaging (N2 to N0–1) compared to the control group (55% vs. 10%, p<0.001), supporting the role of induction therapy in improving nodal status and surgical eligibility.

A clear shift in mediastinal lymph node status, from clinically positive (cN2) to lower pathological stages (ypN0-1), was identified in more than half of the patients treated with induction therapy (22 cases; 55%). This improvement was significantly more pronounced when compared to the group managed with primary surgery (p<0.001) (Figure 2).

In addition, analysis of the resected specimens demonstrated a substantially reduced extent of viable tumor tissue in patients who received neoadjuvant treatment. This finding supports the presence of a strong tumor-reducing effect, which was statistically confirmed (p<0.001).

Predictors of surgical operability

The forest plot presents results of multivariable logistic regression analysis identifying factors independently associated with surgical operability in patients with stage IIIA non-small cell lung cancer. The regression model included tumor regression and nodal downstaging. Effect sizes are expressed as odds ratios (OR) with corresponding 95% confidence intervals (CI).

**Figure 3:**
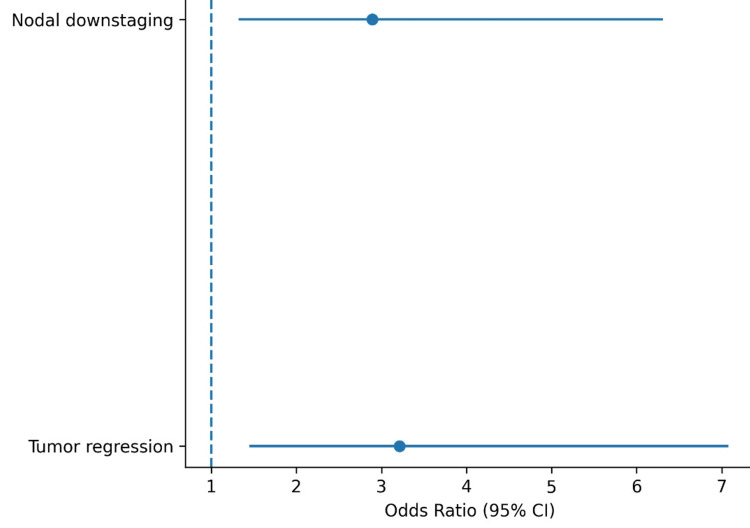
Predictors of surgical operability The forest plot displays odds ratios (OR) with 95% confidence intervals (CI) from multivariable logistic regression analysis. Tumor regression and nodal downstaging were identified as independent predictors of surgical operability.

Both tumor regression and nodal downstaging were identified as independent predictors of a higher likelihood of achieving surgical resection (Figure 3).

## Discussion

The optimal management of stage IIIA NSCLC continues to represent a complex clinical challenge due to the marked heterogeneity of this disease entity. Variability in tumor burden, extent of mediastinal lymph node involvement, and underlying tumor biology necessitates a highly individualized therapeutic approach within a multidisciplinary setting [1-4].

In recent years, increasing attention has been directed toward the role of induction therapy as part of a multimodal treatment strategy. The rationale behind this approach lies in its potential to reduce tumor volume, facilitate surgical resection, and improve long-term outcomes. Several clinical trials and observational studies have demonstrated that preoperative therapy can enhance resectability in selected patients, supporting its growing use in routine practice [5-7].

The findings of the present study further reinforce the importance of treatment response as a determinant of surgical feasibility. Patients who received neoadjuvant therapy showed more favorable pathological outcomes, including higher rates of tumor regression and complete (R0) resection. These observations are consistent with previously published data indicating that response to induction therapy correlates with improved surgical and oncological outcomes [8-10].

A particularly relevant finding of this study is the strong association between mediastinal nodal downstaging and operability. The prognostic significance of nodal response has been well documented, with multiple studies reporting superior survival in patients achieving clearance of mediastinal disease following induction therapy [11-17]. In line with these reports, our results demonstrate a significantly higher rate of nodal downstaging in the neoadjuvant group. Furthermore, multivariate analysis identified nodal status as an independent predictor of complete resection, emphasizing its central role in guiding surgical decision-making [18,19].

Beyond nodal involvement, the degree of pathological tumor regression and the extent of residual viable tumor also emerged as key factors influencing operability. Patients exhibiting major pathological response, particularly those achieving complete response, were more likely to undergo successful anatomical resection. This observation aligns with existing evidence suggesting that pathological response serves as a surrogate marker for improved disease-free and overall survival [20-22]. Consequently, quantitative assessment of residual tumor burden may provide additional value in evaluating treatment efficacy and selecting candidates for surgery [23,24].

Importantly, the incorporation of neoadjuvant therapy did not result in increased perioperative risk in our cohort. Postoperative morbidity and short-term mortality were comparable between groups, despite the more advanced baseline disease characteristics in patients receiving induction treatment. Similar observations have been reported in patients undergoing major resections, including pneumonectomy, after induction therapy, without a significant rise in mortality or complication rates [25]. These findings indicate that surgery can be performed safely following neoadjuvant treatment when appropriate selection criteria and perioperative care are applied.

The clinical implications of these findings are significant. First, they support the integration of tumor regression and nodal downstaging as objective parameters in the assessment of surgical operability. Second, they highlight the necessity of accurate restaging using both advanced imaging modalities and invasive mediastinal staging techniques prior to surgical intervention [26]. Finally, the results of this study further strengthen the concept that surgery remains an essential component of multimodal therapy in carefully selected patients who demonstrate a favorable response to induction treatment.

This study has several limitations, including its retrospective design and relatively small sample size, which may limit generalizability. In addition, potential selection bias can not be excluded due to the non-randomized nature of treatment allocation.

## Conclusions

Tumor response to neoadjuvant therapy, particularly in terms of primary tumor regression and mediastinal nodal downstaging, plays a crucial role in determining surgical operability in patients with stage IIIA NSCLC. In this study, induction therapy was associated with improved resectability and higher rates of complete (R0) resection, without increasing perioperative morbidity or mortality.

These findings support the use of treatment response as a practical parameter in selecting candidates for surgery within a multidisciplinary setting. A tailored multimodal approach that integrates systemic therapy and surgical management may optimize patient selection and improve oncological outcomes. Further prospective studies are needed to validate these findings and refine the criteria for surgical decision-making.
